# Tumour Seeding Following Core Biopsy of a Mucoepidermoid Carcinoma of the Parotid: Radiological Evidence and a Surgical Approach to En-Bloc Dissection of the Mass and Tract

**DOI:** 10.7759/cureus.25715

**Published:** 2022-06-07

**Authors:** Thomas Ringrose, Benjamin M Olley, Leyla Ozbek, Yinan Zhu, Jonathan Hughes

**Affiliations:** 1 Head and Neck Surgery, University College London Hospitals NHS Foundation Trust, London, GBR

**Keywords:** tumour seeding, radiological diagnosis, parotid surgery, mucoepidermoid, tumour

## Abstract

Tumour seeding along the needle tract following core needle biopsy of the parotid is a recognised complication. We present a unique case of mucoepidermoid carcinoma of the parotid in an 18-year-old patient with associated tumour seeding within the core needle biopsy tract. Tumour seeding was confirmed both histologically and radiologically on magnetic resonance imaging as early as 35 days post-biopsy. The patient was treated successfully with a combination of surgery and adjuvant proton beam therapy. This case also visually demonstrates a surgical approach to en-block excision of the mass and tract.

## Introduction

Mucoepidermoid carcinoma represents the most common type of parotid malignancy [1]. Clinical presentation can vary but usually presents with a painful lump in the fifth decade of life with a slight predominance in females [1]. Core needle biopsy (CNB) and fine-needle aspiration cytology (FNAC) remain established techniques in the diagnosis of head and neck masses [2]. Tumour seeding along the needle tract is a recognised but rare complication following core needle biopsy (CNB) of parotid lesions [3].

This report describes a confirmed case of core needle biopsy seeding from a mucoepidermoid carcinoma of the parotid in a young patient, including confirmatory imaging and histology. To the author's knowledge, there is no radiological evidence of tumour seeding post-core needle biopsy of the parotid in the published literature. This case also visually demonstrates a surgical approach to en-bloc excision of the mass and tract.

## Case presentation

An 18-year-old female presented with a six-month history of a non-painful fluctuant swelling overlying the right parotid gland, with no facial weakness. The patient was a non-smoker, had no other co-morbidities, and only took a combined contraceptive pill. An ultrasound of the neck demonstrated a 15x14x10 mm, well-defined, homogeneously hyperechoic lesion within the superficial portion of the right parotid gland, suggestive of a pleomorphic adenoma. A 6 mm diameter intra-parotid lymph node was noted lying superiorly within the superficial lobe, non-pathological in appearance. The rest of the parotid gland appeared normal. Two 1 cm long ultrasound-guided core biopsies were obtained using 18-gauge cutting biopsy needles. Histopathological testing and fluorescent in situ hybridisation (FISH) testing for the MAML2 gene was performed. Morphological features, supported by FISH findings, were those of mucoepidermoid carcinoma. There were no high-grade features in the biopsy.

**Figure 1:**
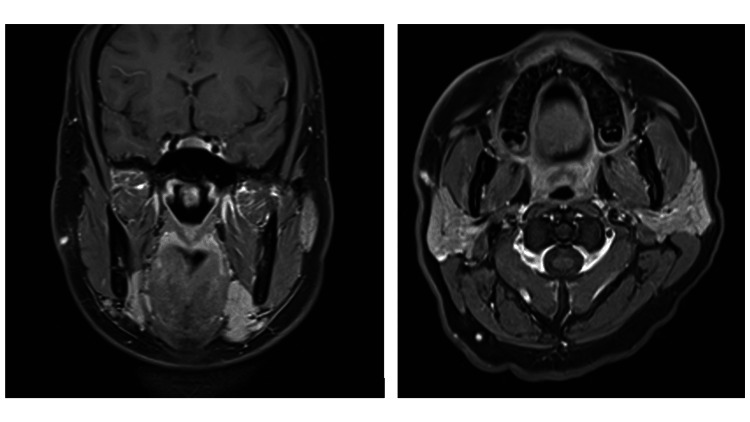
MRI imaging demonstrating seeding through the tract following core biopsy Left image: coronal view; Right image: axial view

Subsequent contrast-enhanced computed tomography (CT) of the neck and chest and magnetic resonance imaging (MRI) of the neck were performed 35 days later. CT imaging demonstrated a 3 mm indeterminate subpleural nodule within the right lower lobe, unlikely to represent metastasis. Follow-up CT imaging in three months was recommended. The MRI demonstrated a 1.5 cm enhancing nodule in the superficial lobe of the right parotid gland and a linear tract to the skin surface, suspicious for local tumour extension (Figure 1). Repeat ultrasound and fine-needle aspiration (FNA) were performed on the tract. Cytology results were acellular and insufficient for diagnosis.

**Figure 2:**
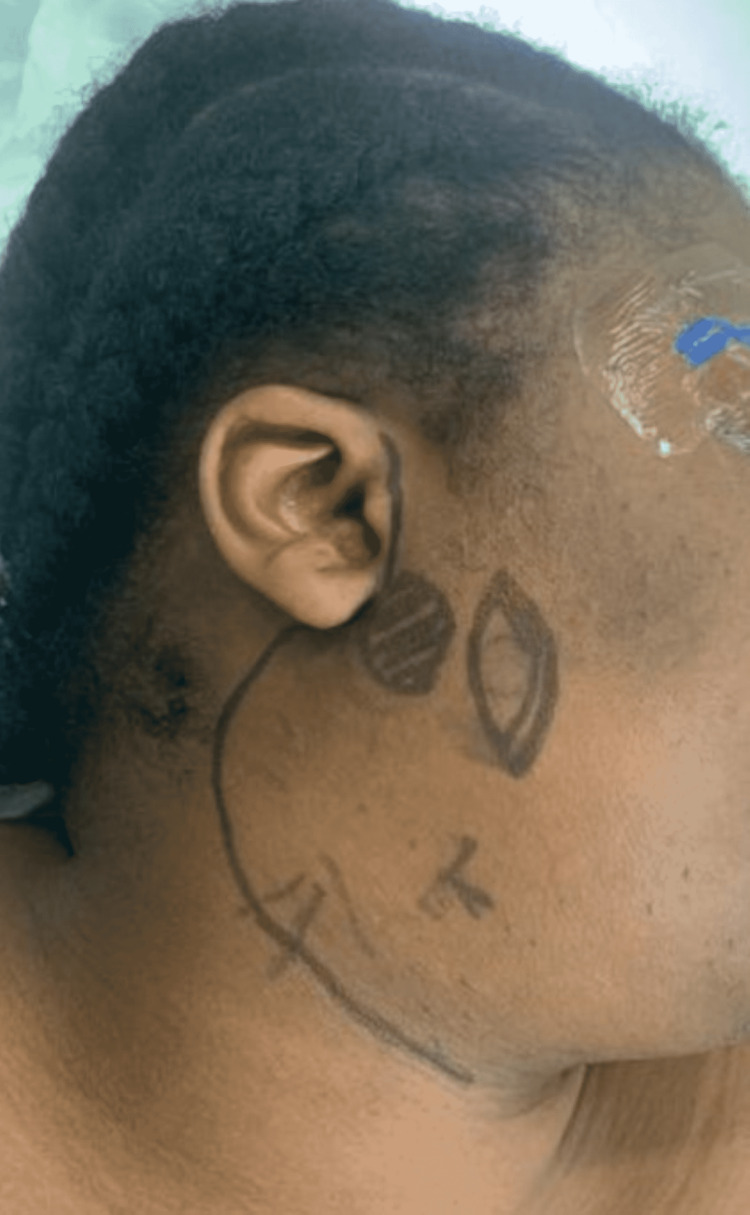
Preoperative surgical planning markings The black shaded area represents the focus of the disease, and the elliptical shape contains an abnormal area of puckered skin through which the CNB was taken. CNB: core needle biopsy

**Figure 3:**
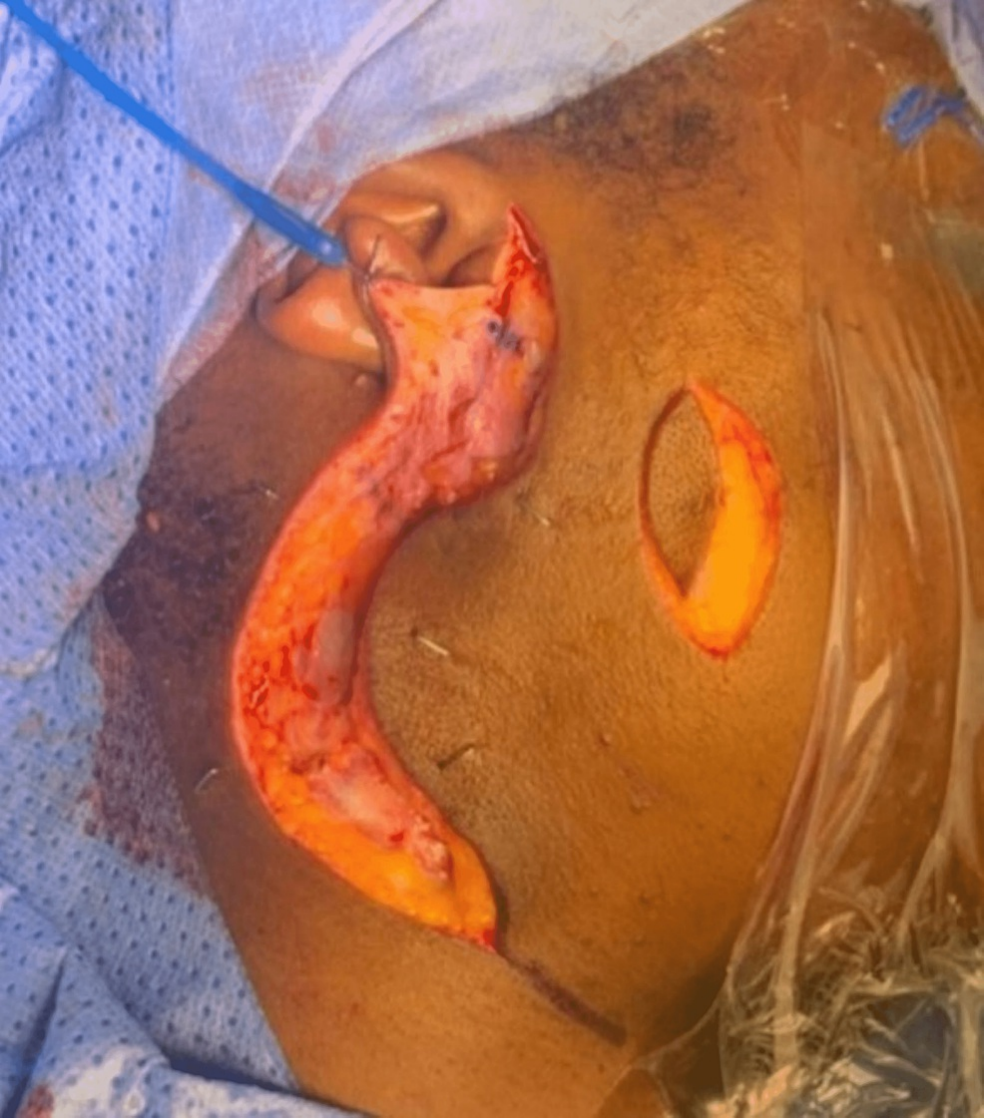
Skin incisions: vertical elliptical incision of abnormal skin and modified Blair incision

**Figure 4:**
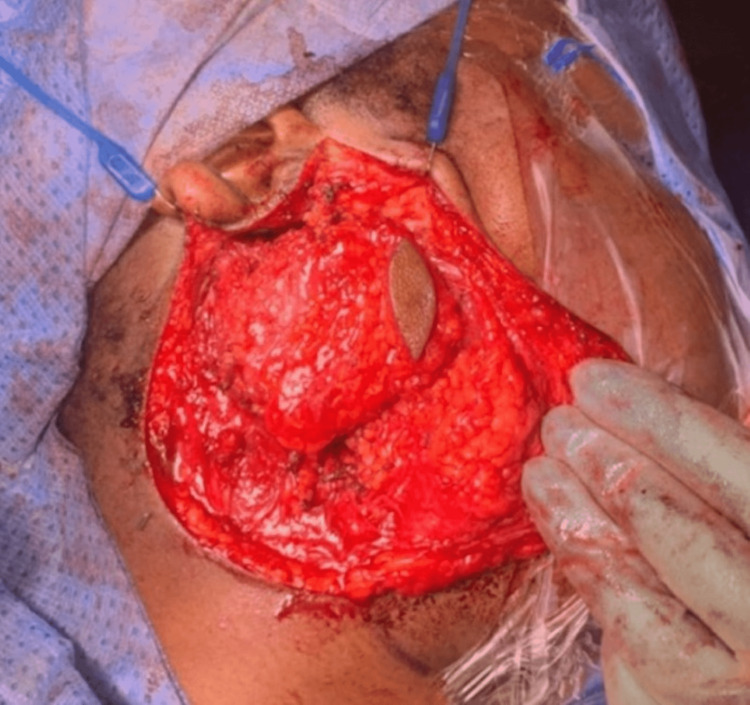
Subplatysmal and suprascapular flaps were raised up to and beyond the anterior edge of the elliptical incision

Surgery was performed to remove the mucoepidermoid carcinoma tumour and associated seeding to the skin from CNB en-bloc with 5 mm margins. The patient was positioned, prepped, and draped in the usual manner with facial nerve monitoring. Surgical planning markings can be seen (Figure 2); the black shaded area represents the focus of the disease and the elliptical shape contains an abnormal area of puckered skin through which the CNB was taken. Two skin incisions were performed - a vertical elliptical incision of abnormal skin and a modified Blair incision (Figure 3). Subplatysmal and suprascapular flaps were raised up to and beyond the anterior edge of the elliptical incision (Figure 4). The rest of the procedure was completed in the usual manner, identifying the facial nerve and dissecting each branch from the specimen.

**Figure 5:**
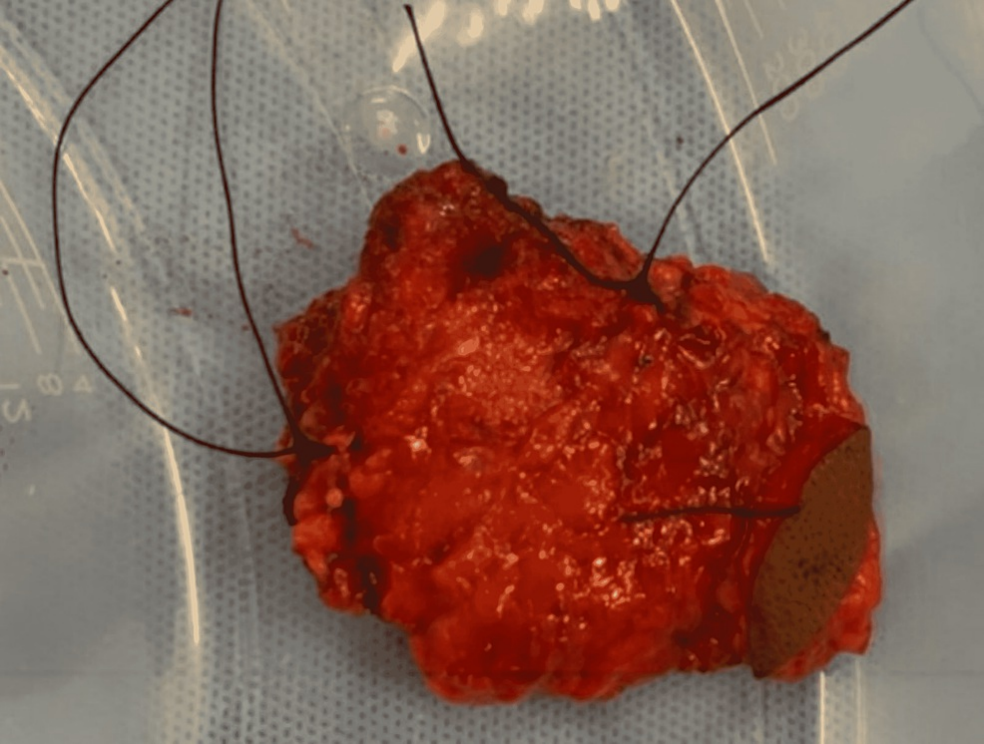
The parotid gland and associated skin abnormality remain intact

The parotid gland and associated skin abnormality remain intact (Figure 5) and were sent for histological analysis. Histopathology demonstrated a confirmed diagnosis of mucoepidermoid carcinoma with two foci of disease. The first was 13x9x6 mm within the parotid parenchyma and staged PT1N0 with a close deep margin. The second focus was within the subcutaneous adipose tissue and was 9x9x5 mm; this was also staged PT1N0. A decision was made for no further surgical intervention following the close margins due to the potential risk to the facial nerve. Adjuvant treatment was recommended, and the patient underwent proton beam therapy instead of radiotherapy in order to reduce the risk of side effects.

## Discussion

The most common type of parotid malignancy is mucoepidermoid carcinoma. It most frequently presents as a painful lump in the fifth decade of life with a slight predominance in females [1]. This case demonstrates that the clinical presentation of mucoepidermoid carcinoma can be varied. This presentation was slightly atypical since the patient was 18 years old with no environmental risk factors and presented with a painless mass and ultrasound findings consistent with pleomorphic adenoma. This hence emphasises the need for histological diagnosis in the workup for patients presenting with parotid masses even in low-risk patients.

Core needle biopsy (CNB) and fine-needle aspiration cytology (FNAC) remain established techniques in the diagnosis of head and neck masses [2]. Numerous studies have shown CNB to yield both greater sensitivity and specificity than FNAC in the diagnosis of head and neck cancers [4-5]. Seeding along the needle tract is a known risk of these procedures and was first described in the 1930s by Martin and Ellis [3]. The incidence of tumour seeding is known to be increased following CNB with some estimates, suggesting rates of tumour seeding in head and neck malignancy to be 0.00012% and 0.0011% after FNA and core needle biopsy, respectively [2]. Other factors associated with increased risk of tumour seeding include the needle gauge, number of passes, and use of suction or capillary techniques.

Reviewing the literature, tract seeding is frequently reported in the assessment of breast malignancy and these commonly present late postoperatively with a palpable mass. A published case report shows MR imaging of a tract seeding preoperatively with breast malignancy [6]. The case we have described is unique, as there are no images in the literature of tumour seeding in the head and neck and changes are seen as early as 35 days post-CNB and this is clinically correlated with subtle skin puckering on examination. General surgical oncological principles dictate that en-bloc resection is preferable for histological analysis so long as it does not increase the risk of the procedure [7]. This could be achieved in this case with a modified flap with an elliptical incision to facilitate a primary closure.

Adjuvant treatment in the form of proton beam therapy (PTB) can be used as an alternative to intensity-modulated radiation therapy (IMRT) for patients with salivary gland tumours, as was the case with this patient. Some studies including a 2020 study by Chuong et al. have recommended that PBT be strongly considered when ipsilateral radiation therapy is indicated for major salivary gland cancer due to a significantly lower incidence of acute toxicity and hence side effects [8].

## Conclusions

In conclusion, we highlight the importance of histological diagnosis in the investigation of patients presenting with parotid masses even in low-risk patients. This case demonstrates, to our knowledge, the only radiological evidence of tumour seeding post-core needle biopsy of a parotid mass. It also visually and neatly demonstrates a general principle of en-block oncological resection when two foci of diseases are involved.
